# The NIST SPIDER, A Robot Crane

**DOI:** 10.6028/jres.097.016

**Published:** 1992

**Authors:** James Albus, Roger Bostelman, Nicholas Dagalakis

**Affiliations:** National Institute of Standards and Technology, Gaithersburg, MD 20899

**Keywords:** cables, kinematically constrained, parallel link manipulator, robot cranes, six degrees of freedom, six meter model, Stewart platform, work platform

## Abstract

The Robot Systems Division of the National Institute of Standards and Technology has been experimenting for several years with new concepts for robot cranes. These concepts utilize the basic idea of the Stewart Platform parallel link manipulator. The unique feature of the NIST approach is to use cables as the parallel links and to use winches as the actuators. So long as the cables are all in tension, the load is kinematically constrained, and the cables resist perturbing forces and moments with equal stiffness to both positive and negative loads. The result is that the suspended load is constrained with a mechanical stiffness determined by the elasticity of the cables, the suspended weight, and the geometry of the mechanism. Based on these concepts, a revolutionary new type of robot crane, the NIST SPIDER (Stewart Platform Instrumented Drive Environmental Robot) has been developed that can control the position, velocity, and force of tools and heavy machinery in all six degrees of freedom (*x, y, z*, roll, pitch, and yaw). Depending on what is suspended from its work platform, the SPIDER can perform a variety of tasks. Examples are: cutting, excavating and grading, shaping and finishing, lifting and positioning. A 6 m version of the SPIDER has been built and critical performance characteristics analyzed.

## 1. Introduction

**Fig. 1:**
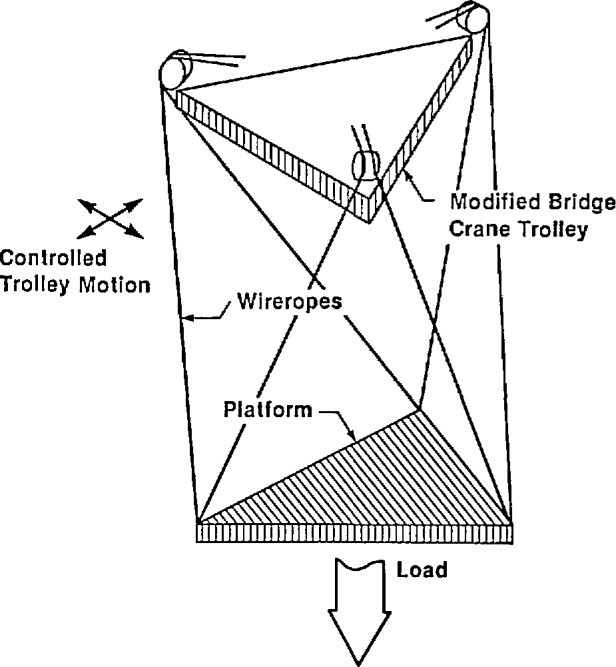
Design concept for improving stiffness of crane suspension mechanisms.

**Fig. 2:**
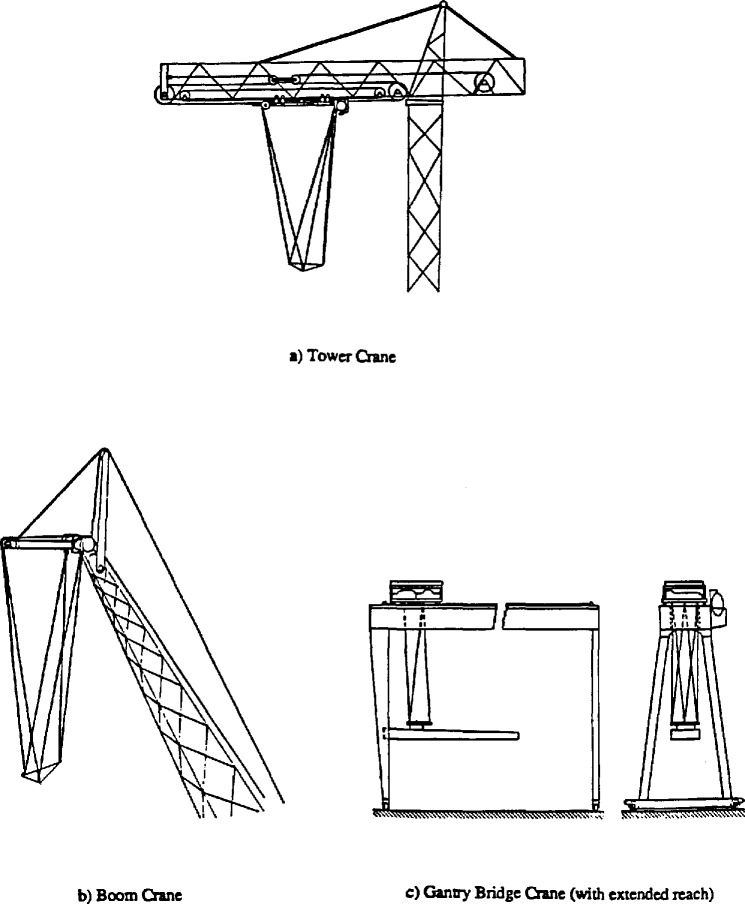
Stabilized platform concept applied to various crane designs.

A new crane design utilizing six cables to suspend a load platform was first developed by the National Institute of Standards and Technology in the early 1980s. A Defense Advanced Research Projects Agency (DARPA) sponsored program on robot crane technology produced the design, development and testing of three different sized prototypes to determine the performance characteristics of this proposed robot crane design. A description of the overall DARPA program and the results of this research are presented in [1]. Initial testing of these prototypes showed that this design results in a stiff load platform [2,3]. This platform (see Fig. 1) can be used in typical crane operations, or as a robot base, or a combination of both. Applications of this new crane design in the construction industry are illustrated in Fig. 2.

**Fig. 3:**
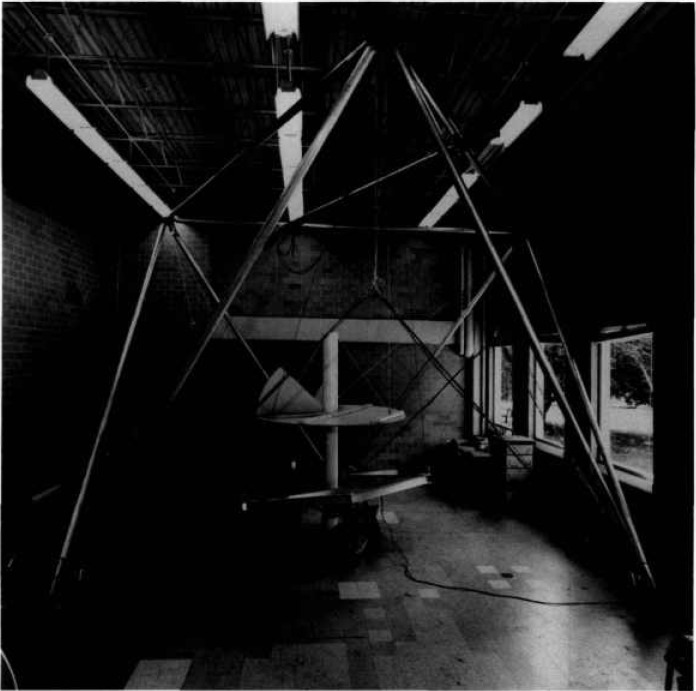
Photograph of the NOWFFR before it was modified to become the SPIDER. Centered is a heat shield/chimney and a chain saw attached to the platform and ready to cut an oak log.

In response to the oil fires set in Kuwait during the Persian Gulf War, NIST adapted its robot crane technology for assisting in extinguishing oil well fires. A system called the NIST Oil Well Fire Fighting Robot (NOWFFR) was designed and constructed. This robot is described in a video tape and a NIST internal working document. A photograph of the NOWFFR is shown in Fig. 3.

**Fig. 4:**
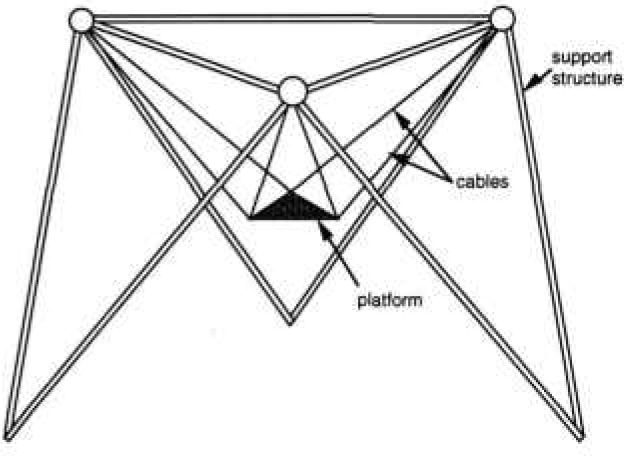
The SPIDER geometry.

Early in 1991, the NOWFFR design was modified for applications related to nuclear and toxic waste site cleanup. The new robot design is called the SPIDER (Stewart Platform Instrumented Drive Environmental Robot). The SPIDER is light weight and easily assembled. The SPIDER can be made mobile by attaching vehicles to the feet so that it can be driven over rough terrain. A conceptual diagram of the SPIDER is shown in Fig. 4.

Two models of the SPIDER have been designed and constructed. A 2 m model has been used to test mobility issues. A 6 m model has been used to test lifting and load positioning perameters and to analyze the size and shape of the work volume.

## 2. Objective

The objective of this paper is to report on the design, development and evaluation of the SPIDER. The primary function of the SPIDER is to lift, maneuver, and position large loads with precise control of position and force in all six degrees of freedom.

The SPIDER consists of a stable platform supported by six cables suspended from three points on a fixed or mobile octahedral structure. The six cables are arranged to kinematically constrain the stable platform such that its stiffness is determined by the tensile elasticity of the cables. Maximum stiffness is maintained so long as perturbing forces and/or torques are below a threshold determined by the weight of the load. For forces or torques above that threshold, one or more cables will go slack, and stiffness will drop to that generated by pendulum forces of the load on the cables remaining taut.

When all six cables are in tension, the stable platform is kinematically constrained, and there exists a known mathematical relationship between the lengths of the six cables and the position and orientation of the platform. The theory of this mathematical relationship has been known for many years. The theory was first embodied in a Stewart platform for testing tires in the 1950s [4], and applied to aircraft flight simulators during the 1960s and 1970s. It was applied to cable driven manipulators by Landsberger [5,6] and to cranes by NIST in the 1980s [1].

On the SPIDER, the lengths of the six cables are controlled by six winches. These are controlled and coordinated by a computer. Input commands from a six-axis joystick enable an operator to control the motion of the stable platform in all six degrees of freedom (*x, y, z*, roll, pitch, and yaw). The operator can thus manuever the stable platform, and whatever load or tool that is attached to it, over a large working volume.

The three support points of the octahedron are carried by three vehicles for mobility. Coordination of vehicle track motions to accomplish steering and velocity commands provided by an operator is also done by computer.

## 3. Structure and Control

The framework of the SPIDER is a six legged structure resting on three support points. The legs are 6 m aluminum tubes that are 10 cm in diameter and constructed in an octahedron geometry. These can be seen in Fig. 3, which is a photograph of the NOWFFR taken before it was modified to become the SPIDER. The top of the SPIDER structure consists of a triangle. Each vertex of the triangle supports two cables. Together, the six cables support a lower work platform. The connecting joints at the vertices consist of ball and socket joints. Due to its octahedron geometry, all forces are directed through points at the vertices. As a result there are no bending or twisting moments generated by the load. Each member of the structure is always in pure compression or tension except for supporting its own weight. The SPIDER structure thus provides near maximum strength and stiffness possible for any given mass of structural material.

**Fig. 5:**
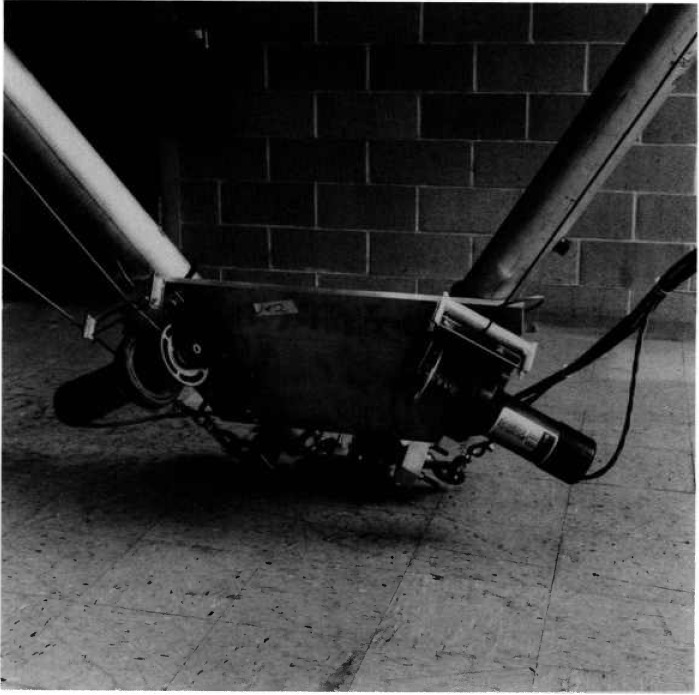
One of three pairs of winches used to maneuver the platform.

The lower work platform is supported by six braided steel cables 5 mm in diameter. The length of each cable is controlled by a winch having a 455 kg load rating. Figure 5 shows the winches. The cables run from the winches up and over pulleys at the vertices of the upper triangle, and back down to the lower work platform. By controlling the six winches, an operator can maneuver the lower work platform in six degrees of freedom. The work platform is made of aluminum I-beams. It can be a variety of sizes. Studies at NIST have shown that a 2 to 1 ratio of the size of the upper triangle to the lower platform is the most stiff for lateral force loading [2].

**Fig. 6:**
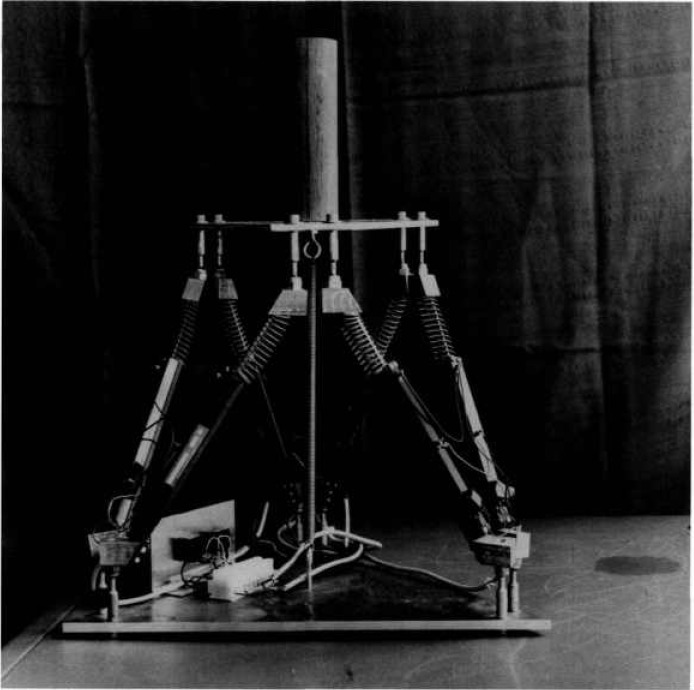
Stewart platform joystick used as operator interface to drive the lower platform.

**Fig. 7:**
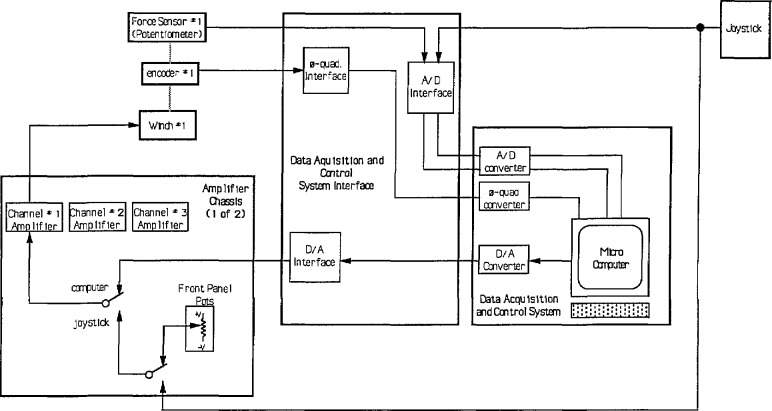
Control system architecture for the SPIDER showing one of six channels.

The joystick used to control the SPIDER is a Stewart Platform with linear potentiometers as shown in Fig. 6. By orienting this joystick so that the potentiometers are roughly parallel to the SPIDER cables, the control mode can be master-slave rate control. In this mode, the velocity of each winch is controlled by the displacement of its corresponding potentiometer. Over a limited range wherein the cables and potentiometers remain roughly parallel, the SPIDER motion can be controlled directly by the potentiometers without a computer. A diagram of this control mode is shown in Fig. 7. The joystick potentiometers generate analog voltages for amplifiers that control the winches. This is called the Manual mode.

The SPIDER also has a Computed-Manual mode. In this mode the joystick signals are switched to send analog voltages from the joystick to a computer. Joystick potentiometers signals are fed into an analog-to-digital (A/D) board embedded in the computer. From these signals the computer calculates the joystick input position and orientation and translates it to SPIDER command cable lengths. Feedback is received from cable length and force sensors located near the winches. This enables closed loop position, velocity, and force control. Cable travel encoders generate phase quadrature signals for an encoder input board embedded in the computer. Cable tension sensors are input into an A/D board in the computer. Command signals are output from the computer via a digital to analog (D/A) board and sent to the winch amplifiers.

Control algorithms being developed for the SPIDER include forward and reverse kinematics that are necessary to allow trajectory control of the lower platform. With trajectory control, the work platform could be made to follow a particular path while the computer interprets joystick information to control the speed of the platform.

## 4. Comparison to Conventional Cranes

Existing cranes of many different types from many manufacturers, are able to lift comparable loads, but cannot stabilize the loads in rotation or sway. Under ideal conditions, a highly skilled crane operator can provide some measure of oscillation damping. However, for precise orientation, a crew of riggers is needed to manually stabilize the load from rotating and swinging and to manually guide the load into its final desired position.

Existing cranes, even with expert operators, cannot prevent perturbations such as wind from causing the load to sway, in some cases by more than a meter. Novice operators of conventional cranes may have difficulty in preventing heavy loads from colliding with objects in the environment. Existing cranes provide little or no load stabilization against rotations, and have no means of controlling forces or torques on the load.

The principal advantage of the SPIDER is that it provides sufficient control to allow even a novice operator to position a load without sway to within a few millimeters in *x, y*, and z, and to control orientation without oscillation to within 1° in roll, pitch, and yaw. Force sensors on the SPIDER winch mechanisms could also allow the operator (with computer assistance) to control forces and torques on a load after it comes into contact with the environment. The control provided by the SPIDER could thus reduce the size of the crew needed to manually position loads from three or four, to zero or one.

An additional advantage of the SPIDER is its high lift-to-weight ratio. Due to its octahedron geometry, the SPIDER requires no counter weight and experiences no twisting or bending moments. As a result, it can lift at least five times its own weight. This is significantly more than any robot or crane in current use.

## 5. Applications

The principal applications of the SPIDER are expected to be lifting and positioning objects or power tools weighing up to a ton. Depending on what is suspended from its work platform, the SPIDER could perform a variety of tasks that are not possible by conventional cranes. For example,

### For cutting

The SPIDER could manipulate a variety of saws (chain saw, wire saw, or disc saw), rotary cutting tools (router, milling tool, grinding tool), abrasive jet tools (water jet, air jet), flame cutters, or chisels (jack hammer, sculpting chisel, etc.) for cutting concrete, steel, wood, or stone. The SPIDER can produce large forces with accuracies sufficient for many types of machining operations, including milling, routing, drilling, grinding, and polishing. SPIDER motions could be controlled manually, or by a computer such as currently used for numerically controlled machine tools.

### For excavating and grading

The SPIDER could manipulate digging devices (ditching or trenching machines, digging tools, augers, scrapers) precisely over the ground in either a manual or computer controlled mode. Dirt, stone, concrete, or asphalt could be removed from a large volume with great precision. The robot can easily manuever loads of several tons. This implies that the SPIDER work platform could carry a gasoline or diesel engine, power transmission system, and tooling for excavating and grading. The SPIDER could also carry a large bucket for removing soil and loading it in trucks or conveyors. SPIDER motions could be controlled manually, or automatically based on data bases generated by computer aided design systems.

### For shaping and finishing

The SPIDER could manipulate grinders, polishers, buffers, paint sprayers, sandblasters, and welding torches over large objects (ship hulls, structural steel, castings and weldments, concrete structures). It can apply controlled amounts of force and resist perturbations in all directions. Motions could be controlled manually, or automatically from computer data base models of objects.

### For lifting and positioning

The SPIDER could be fitted with a variety of gripping devices to lift and precisely position heavy loads such as concrete or steel beams and pillars. The SPIDER can exert controlled forces to mate and seat loads and can resist perturbations such as wind and inertial forces. Precision motions of 2 mm and 0.5° of rotation can easily be achieved while manuevering large loads.

### For flexible fixturing

The SPIDER work platform is stiff enough to serve as a fixture for holding parts during assembly or construction operations. Parts weighing up to a ton can be held rigidly to resist or exert lateral forces equivalent to half the weight of the load. The SPIDER can also resist or exert torques.

### For transporting manipulators

The SPIDER stable platform can be used as a stable mobile base to support robotic or teleoperated manipulators. For example, manipulator arms mounted on the SPIDER can be used for handling toxic or radioactive waste or for cleaning-up waste sites. The mobility of the SPIDER support structure allows it to position itself over a waste site while keeping its support wheels away from the contaminated soil.

### For fighting oil well fires

The robot could be used to position a chimney and fire shield, to clear debris and excavate around the well head, and to manipulate tools and valves to extinguish the fire and bring the well under control.

## 6. Limits of Workspace Study

The term “limits of the workspace” here is meant to refer to those poses of the robot lower platform at which the tension in one or more of the suspension cables become zero. An undesirable result of this situation is that the robot controller loses control over a corresponding number of degrees of freedom.

### 6.1 Mathematical Model

**Fig. 8:**
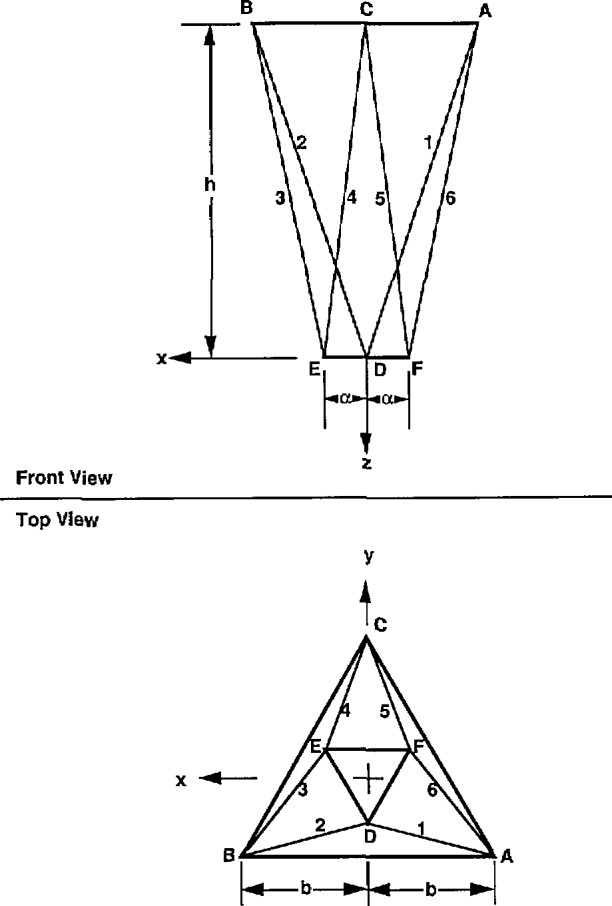
Robot crane cable support structure.

The basic structure of the robot crane, which consists of the cable support system, is shown in Fig. 8 for the resting steady state position. The overhead support and the suspended platform are represented by two equilateral triangles. In this position both triangles are assumed to be horizontal with their centers of gravity lying on the vertical axis *z*. The overhead triangle is assumed to be fixed in space and has three vertices located at

$$\begin{array}{l} \bar{A}:\left(-b,-b\surd \bar{3}/3,-h\right)\\ \bar{B}:\left(b,-b\surd \bar{3}/3,-h\right),\\ \bar{C}:\left(0,2b\surd \bar{3}/3,-h\right). \end{array}$$ (1)

with respect to a Cartesian coordinate frame (*x,y,z*), centered at the center of gravity of the lower triangle, when it is positioned at its steadystate, resting-state home pose (see Fig. 8). *2b* is the length of the side of the overhead triangle and *2α* isWhere *Q_ij_* is the *i*th row, *j*th column element of matrix *Q*

$$\bar{Q}=\left[\begin{array}{lll} \cos\Psi \;\cos\phi -\sin\Psi \;\sin\theta \;\sin\phi & -\cos\theta \;\sin\phi & \sin\Psi \;\cos\phi +\cos\;\sin\theta \sin\phi \\ \cos\Psi \;\sin\phi +\sin\Psi \;\sin\theta \;\cos\phi & \cos\theta \;\cos\phi & \sin\Psi \;\sin\phi -\cos\Psi \;\sin\theta \;\cos\phi \\ -\sin\Psi \;\cos\theta \; & \sin\theta \; & \cos\Psi \;\cos\theta \; \end{array}\right]$$

The vectors *l_n_*′ (n = 1,…,6) defining the new position of the wires can be found from Eqs. (1) and (3) and are expressed as

$$\begin{array}{l} {\bar{l}}_{1}\prime =\bar{A}-\bar{D}\prime =\left(-b+2aQ_{12}\surd \bar{3}/3-u_{x},-b\surd \bar{3}/3+2aQ_{22}\surd \bar{3}/3-u_{y},-h+2aQ_{32}\surd \bar{3}/3-u_{z}\right)\\ {\bar{l}}_{2}\prime =\bar{B}-\bar{D}\prime =\left(b+2aQ_{12}\surd \bar{3}/3-u_{x},-b\surd \bar{3}/3+2aQ_{22}\surd \bar{3}/3-u_{y},-h+2aQ_{32}\surd \bar{3}/3-u_{z}\right)\\ {\bar{l}}_{3}\prime =\bar{B}-\bar{E}\prime =\left(b-aQ_{11}-aQ_{12}\surd \bar{3}/3-u_{x},-b\surd \bar{3}/3-aQ_{21}-aQ_{22}\surd \bar{3}/3-u_{y},-h-aQ_{31}-aQ_{32}\surd \bar{3}/3-u_{z}\right)\\ {\bar{l}}_{4}\prime =\bar{C}-\bar{E}\prime =\left(-aQ_{11}-aQ_{12}\surd \bar{3}/3-u_{x},2b\surd \bar{3}/3-aQ_{21}-aQ_{22}\surd \bar{3}/3-u_{y},-h-aQ_{31}-aQ_{32}\surd \bar{3}/3-u_{z}\right)\\ {\bar{l}}_{5}\prime =\bar{C}-\bar{F}\prime =\left(aQ_{11}-aQ_{12}\surd \bar{3}/3-u_{x},2b\surd \bar{3}/3+aQ_{21}-aQ_{22}\surd \bar{3}/3-u_{y},-h+aQ_{31}-aQ_{32}\surd \bar{3}/3-u_{z}\right)\\ {\bar{l}}_{6}\prime =\bar{A}-\bar{F}\prime =\left(-b+aQ_{11}-aQ_{12}\surd \bar{3}/3-u_{x},-b\surd \bar{3}/3+aQ_{21}-aQ_{22}\surd \bar{3}/3-u_{y},-h+aQ_{31}-aQ_{32}\surd \bar{3}/3-u_{z}\right) \end{array}$$

the length of the side of the lower triangle. The height *h* is the vertical distance between the two triangles.

The lower triangle, before it is displaced, has three vertices located at

$$\begin{array}{l} \bar{D}:\left(0,-2a\surd \bar{3}/3,0\right),\\ \bar{E}:\left(a,\;a\surd \bar{3}/3,0\right)\\ \bar{F}:\left(-a,\;a\surd \bar{3}/3,0\right) \end{array}$$ (2)

with respect to the same coordinate frame.

Let the lower triangle undergo a rigid body motion characterized by three displacements, *u_x_,u_y_, u_z_*, and three successive rotations performed in the following sequence: first, rotation by an angle *ϕ* about z-axis, then *θ* about x-axis, and then *ψ* about *y*-axis. Then it was shown in [1] that after the end of the motion the new coordinates of the vertices of the lower platform are

$$\begin{array}{l} \bar{D}\prime :\left(-2aQ_{12}\surd \bar{3}/3+u_{x},-2aQ_{22}\surd \bar{3}/3+u_{y},-2aQ_{32}\surd \bar{3}/3+u_{z}\right),\\ \bar{E}\prime :\left(aQ_{11}+aQ_{12}\surd \bar{3}/3+u_{x},aQ_{21}+aQ_{22}\surd \bar{3}/3+u_{y},aQ_{31}+aQ_{32}\surd \bar{3}/3+u_{z}\right),\\ \bar{F}\prime :\left(-aQ_{11}+aQ_{12}\surd \bar{3}/3+u_{x},-aQ_{21}+aQ_{22}\surd \bar{3}/3+u_{y},-aQ_{31}+aQ_{32}\surd \bar{3}/3+u_{z}\right). \end{array}$$ (3)

The balance of forces acting on the lower platform requires that

$$\bar{f}+\sum_{n=1}^{6}{\bar{f}}_{n}=0$$ (6)

where $\bar{f}$ is the external force applied at the center of gravity of the platform. $\bar{f}$ is equivalent to *W* for this case. The directions of the wire tensions ${\bar{f}}_{n}$ are given by the corresponding vectors of Eq. (5).

The balance of moments acting on the lower platform requires that

$$\bar{m}+\bar{Q}\bar{D}\times \left({\bar{f}}_{1}+{\bar{f}}_{2}\right)+\bar{Q}\bar{E}\times \left({\bar{f}}_{3}+{\bar{f}}_{4}\right)+\bar{Q}\bar{F}\times \left({\bar{f}}_{5}+{\bar{f}}_{6}\right)=0$$ (7)

where $\bar{m}$ is the external moment applied upon the lower platform, $\bar{m}$ is zero for the workspace limit study and the only externally applied force is the single load of weight *W* suspended from the centroid of the lower platform.

Two conditions under which two of the cables always become loose for any value of the weight *W* have been identified.

### 6.2 Condition #1

**Fig. 9:**
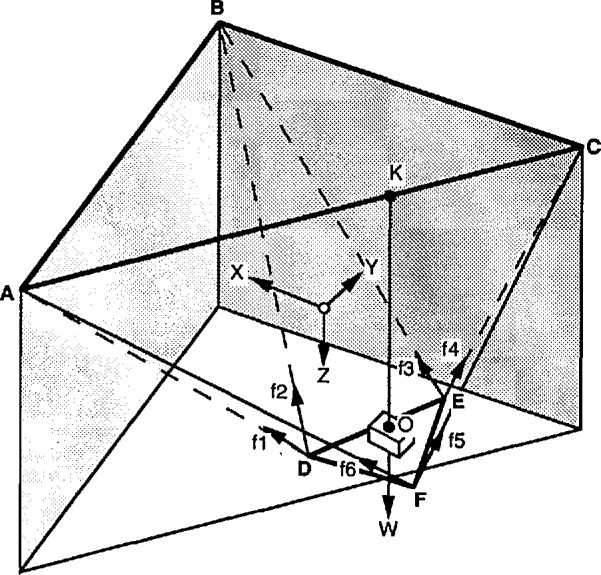
Condition #1–work-volume edge detection.

Figure 9 shows the position of the lower platform under which the tensions in two of its suspension cables (2 and 3) go to zero. For this condition to occur the vector of the weight *W* must cross a vertical plane through one of the suspension lines AC or AB or BC. These suspension lines are defined by the three suspension points A, B, and C. From each of these points two suspension cables originate. So each suspension line supports four cables. For example, in the case of line AC shown in the Fig. 9, cables 1, 4, 5, 6 all intersect a vertical plane that includes AC and only these four cables are in tension.

The sum of the moments of all the cable tensions about *K*, which is the point at which the vector of the weight *W* intersects a vertical plane that includes the suspension line AC (see Fig. 9) is

$$\bar{K}\bar{D}\times \left({\bar{f}}_{1}+{\bar{f}}_{2}\right)+\bar{K}\bar{E}\times \left({\bar{f}}_{3}+{\bar{f}}_{4}\right)+\bar{K}\bar{F}\times \left({\bar{f}}_{5}+{\bar{f}}_{6}\right)=0$$ (8)

The projections of these moment vectors on AC can be calculated by multiplying with *U*_ac_ which is the unit vector defining the orientation of AC.

$$\left\{{\bar{U}}_{\text{ac}}\left(\bar{K}\bar{D}\times {\bar{f}}_{2}+\bar{K}\bar{E}\times {\bar{f}}_{3}\right)\right\}+\left\{{\bar{U}}_{\text{ac}}\left(\bar{K}\bar{F}\times \left({\bar{f}}_{5}+{\bar{f}}_{6}\right)+\bar{K}\bar{D}\times {\bar{f}}_{1}+\bar{K}\bar{E}\times {\bar{f}}_{4}\right)\right\}=0.$$ (9)

But

$$\left\{{\bar{U}}_{\text{ac}}\left(\bar{K}\bar{F}\times \left({\bar{f}}_{5}+{\bar{f}}_{6}\right)+\bar{K}\bar{D}\times {\bar{f}}_{1}+\bar{K}\bar{E}\times {\bar{f}}_{4}\right)\right\}=0.$$ (10)

Since *U*_ac_ is orthogonal to the vector inside the parenthesis. Thus

$$\left\{{\bar{U}}_{\text{ac}}\left(\bar{K}\bar{D}\times {\bar{f}}_{2}+\bar{K}\bar{E}\times {\bar{f}}_{3}\right)\right\}=0.$$ (11)

The only way for Eq. 11 to be true is if ${\bar{f}}_{2}={\bar{f}}_{3}=0$, or cables 1, 2 and 3, 4 to be horizontal or cables 2 and 3 to cross the suspension line AC. Since the controller will never allow any cables to become horizontal or cross a suspension line, ${\bar{f}}_{2}={\bar{f}}_{3}=0$ is the only possible solution. This has to be true for any *W* and feasible platform orientation.

### 6.3 Condition #2

**Fig. 10:**
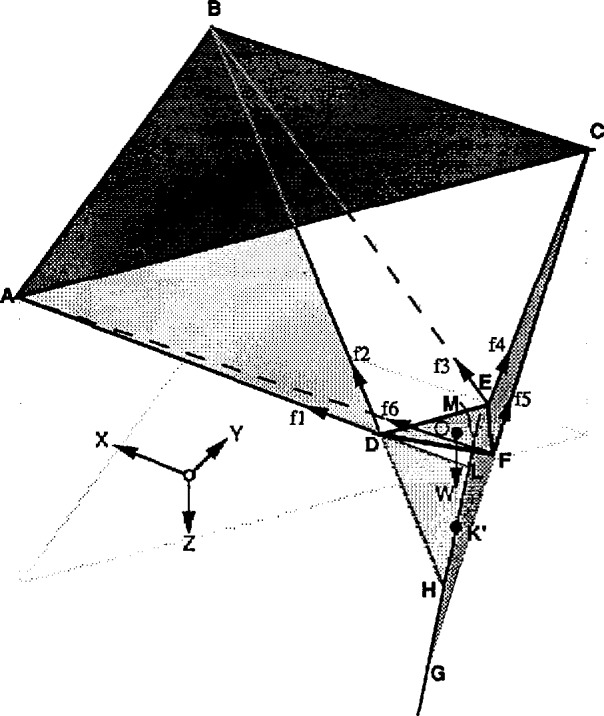
Condition #2–work-volume edge detection.

Figure 10 shows another position of the lower platform under which the tensions in two of its suspension cables go to zero and the corresponding cables become loose (cables 3 and 6). In this case the four-cables crossing line GHLM is an imaginary one defined by the intersection of the planes defined by the two pairs of the active cables (1, 2 and 4, 5). In the case of the Fig. 10, plane ADB and FEC. As soon as the vector of *W* crosses that line cables 3 and 6 become loose. The explanation is the same given in Sec. 6.2.

## 7. Experiments Performed

Experiments were done on the SPIDER to measure payload, work volume, and platform-movement precision. A weight of 455 kg (1000 lb), was maneuvered by the SPIDER platform while in manual mode. The load was carried to the limits of the work volume until cables began to go slack.

As discussed in Sec. 4, two conditions whereby cables begin to go slack can be shown mathematically. As the cables begin to go slack, a point on the edge of the workvolume is defined. Experiments to define this volume were done to verify the mathematics of this phenomena.

Cable loosening conditions #1 and #2 were tested for total platform weights of 68 and 455 kg. The platform was moved to cover a quarter of the workspace at an arc of 90°. For condition #2, strings were attached to the cables to extend their length and locate the approximate position of the four-cables crossing line and its intersection with a plumb-line suspended from the centroid of the platform.

A cable loosening computer simulation program has also been developed which is being used to search for the locus of the platform poses satisfying condition #2.

**Fig. 11:**
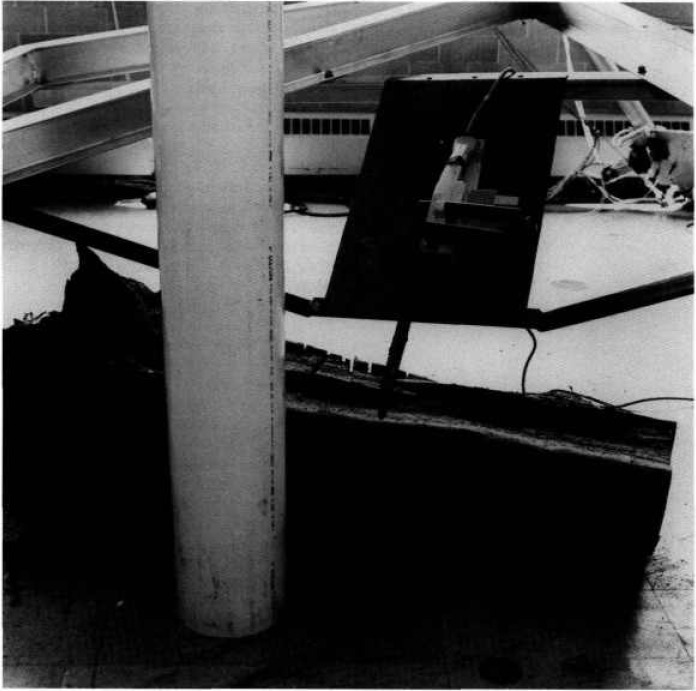
Chain saw mounted on a “leaf-spring” steel plate that is attached to the platform. Note the cuts in the oak log beneath the saw.

Another experiment that was perfomed involved the platform-movement precision and stability. A chain saw was attached to the work platform (see Fig. 11) at a 30° angle from the vertical axis and with the tip of the chain saw blade at the center of gravity of the work platform. The saw was attached to a 3 mm thick steel plate that acted as a leaf-spring. While in the manual mode, depth of cut to within 1 mm could be made in a solid oak log. Deep cuts could also be made with ease either with the blade tangent to, or perpendicular to the oak log surface. With the stability of the platform, little vibration was seen on the chain saw or the steel leaf-spring plate even while driving the tip of the chain saw blade directly into the oak log.

## 8. Summary and Conclusions

The SPIDER robot is a variation of the robot crane design presented in previous reports. Theoretical, computer, and experimental studies of the possible use of this type of robot are given. A preliminary investigation of the limits of its work-space and the current version of its controller are discussed. Several tests were performed to verify the theoretically predicted limits of the work-space.

Work volume measurements verified that cables go slack due to conditions 1 and 2. During this experiment, a payload of 455 kg was carried through the work volume with ease. No strain on the winches was observed while picking up such a load.

The SPIDER can be used for a number of general tasks:

- cutting
- excavating and grading
- shaping and finishing
- lifting and positioning
- flexible fixturing
- transporting manipulators

These capabilities can be targeted for a variety of specific applications such as: fighting oil well fires or hazardous waste site inspection and clean-up.

A number of advantages of the SPIDER over current technology are:

- rigid support and precise maneuverability of large loads
- remote positioning of tools and equipment
- executing precise motions with tools and equipment to accomplish complex tasks
- high lift-to-weight ratio
- resistance to environmental perturbations
- accurate control of loads by a novice operator
- reduced crew size

Future research on SPIDER will include integrating more advanced sensing capability, such as machine vision, and additional mechanical analysis and testing. The long range goal is to build a full-scale working prototype jointly with an industrial partner.
